# Binocular and Fellow Eye Acuity Deficits in Amblyopia: Impact of Fixation Instability and Sensory Factors

**DOI:** 10.3390/jemr18030020

**Published:** 2025-06-03

**Authors:** Yulia Haraguchi, Gokce Busra Cakir, Aasef Shaikh, Fatema Ghasia

**Affiliations:** 1 School of Medicine, Case Western Reserve University, Cleveland, OH 44106, USA; 2 Ocular Motility & Vision Neurosciences Laboratory, Cole Eye Institute, Cleveland Clinic, Cleveland, OH 44106, USA; 3 Daroff-Dell’Osso Ocular Motility Laboratory, Louis Stokes Cleveland VA Medical Center, Cleveland, OH 44106, USA

**Keywords:** binocular visual acuity, fellow eye visual acuity, amblyopia, strabismus, fixation eye movements, fixation instability, nystagmus

## Abstract

Amblyopia, a neurodevelopmental disorder, is commonly assessed through amblyopic eye visual acuity (VA) deficits, but recent studies also highlight abnormalities in the fellow eye. This study quantified binocular and fellow/dominant eye VA in individuals with amblyopia and strabismus without amblyopia and examined factors influencing these measures, including fixation eye movement (FEM) abnormalities. Identifying which subsets of patients—such as those with nystagmus, concurrent strabismus, or greater fixation instability—exhibit more pronounced deficits in binocular visual acuity and binocular summation can enhance clinical decision-making by enabling tailored interventions and aiding patient counseling. Sixty-eight amblyopic, seventeen strabismic without amblyopia, and twenty-four control subjects were assessed using an adaptive psychophysical staircase procedure and high-resolution video-oculography to evaluate FEMs and fixation instability (FI). Binocular and fellow eye VA were significantly lower in amblyopia, regardless of type or nystagmus presence, whereas binocular and dominant eye VA in strabismus without amblyopia did not differ from the controls. Despite reduced binocular acuity, amblyopic and strabismic subjects exhibited binocular summation, with binocular VA exceeding fellow/dominant eye VA. Reduced binocular VA correlated with greater fellow eye VA deficits, diminished binocular summation, and increased FI in the amblyopic eye. Fellow eye VA deficits were linked to greater amblyopic eye VA deficits, an increased degree of anisometropia, higher FI, and stronger nystagmus correlation. These findings suggest amblyopia affects both visual sensory and motor systems, impacting binocular function and fixation stability, with potential consequences for everyday visuomotor tasks like reading.

## 1. Introduction

Amblyopia, a neurodevelopmental disorder, arises from abnormal visual input during early life, commonly due to anisometropia, strabismus, or visual deprivation [1]. This condition leads to reduced grating, vernier, and optotype acuities, as well as decreased contrast sensitivities in the amblyopic eye [2]. Psychophysical studies have demonstrated that the fellow eye is also affected, displaying deficits such as impaired contrast sensitivity, reduced grating acuities, motion perception difficulties, and diminished visual search performance, albeit to a lesser extent than observed in the amblyopic eye [3,4].

Amblyopia also affects visual functions under natural binocular viewing conditions such as reduced stereopsis [5,6], inter-ocular suppression (involving cortical inhibition of perception in one eye’s visual field) [7,8,9], increased fixation instability [7,10], and reduced contrast sensitivity [11,12,13,14]. Further, recent investigations highlight the impact of amblyopia on everyday tasks, such as slow reading and difficulties with visual search [15,16]. Recognizing that binocular visual acuity may offer an accurate representation of visual functions in natural binocular viewing conditions has prompted its consideration as a valuable metric in evaluating amblyopia treatment outcomes [17]. Both patching and binocular treatments have been shown to improve binocular visual acuity [18], with binocular therapies also improving visual function deficits in the fellow eye [19].

The factors influencing binocular visual acuity in amblyopia and strabismus are multifaceted, involving both visual sensory and motor components. Key sensory factors include deficits in the acuity of the fellow eye and amblyopic eye, stereopsis deficits, and the extent of anisometropia [20,21]. Motor factors encompass the presence of strabismus and increased fixation instability, which can arise from nystagmus or alterations in physiologic fixation eye movements (FEMs), such as increased amplitude of fixational saccades and increased inter-saccadic drifts [22,23,24]. Studies have shown that greater fixation eye movement abnormalities are prevalent in amblyopic subjects with poor stereoacuity and more severe visual acuity deficits in the amblyopic eye [25,26,27,28]. Additionally, binocular summation, which integrates input from both eyes to enhance visual performance, is compromised in amblyopia. This summation is typically observed in visual acuity, contrast sensitivity, and fixation stability in normal subjects [13]. However, amblyopic individuals have reduced binocular contrast sensitivity and diminished binocular summation of contrast sensitivity, which has been linked to the depth of amblyopia [11,13,14].

Despite these findings, there remains limited knowledge on the interplay between various visual sensory and motor abnormalities and their collective impact on binocular summation of visual acuity in amblyopia and strabismus. The current study is the first to employ a psychophysical staircase procedure to precisely quantify visual acuities, assess binocular summation of visual acuity, and correlate these measurements with Fixational Eye Movement data. Elucidating the relationship between fixation eye movement (FEM) abnormalities and visual acuity allows us to assess whether the presence of certain characteristics in amblyopia (such as concurrent strabismus, presence of nystagmus, and magnitude of fixation instability) impact the severity of fellow eye and binocular deficits, which can potentiate specialized interventions in different types of amblyopia.

We hypothesize that binocular visual acuity deficits will be observed in amblyopic subjects rather than in those with strabismus without amblyopia, with greater deficits seen in individuals with reduced binocular summation, greater fellow eye visual acuity deficits, and increased fixation instability. Typically, nystagmus is most pronounced during monocular viewing compared to binocular viewing conditions [29]. Additionally, increased fixation instability is observed in binocular viewing conditions in subjects with and without nystagmus. Therefore, we hypothesize that binocular visual acuity deficits will be present in amblyopic subjects regardless of the presence of nystagmus, whereas fellow eye visual acuity deficits will be more pronounced in those with nystagmus.

## 2. Materials and Methods

The Cleveland Clinic Institutional Review Board approved the experimental protocol. Written informed consent was obtained from each participant’s parent/legal guardian in accordance with the Declaration of Helsinki.

### 2.1. Study Cohort

We recruited 68 people with amblyopia (28 with anisometropic amblyopia, 20 with strabismic amblyopia, and 20 with mixed amblyopia), 17 people with strabismus without amblyopia, and 24 control subjects seen in the Pediatric Ophthalmology and Adult Strabismus department of our institution. All participants had comprehensive eye exams, including cycloplegic refraction, ocular motility, strabismus, and stereoacuity measurements. The control subjects were chosen based on the absence of any ocular or systemic abnormalities affecting visual acuity, except for refractive errors. We recruited 17 strabismic subjects without amblyopia or a history of previously treated amblyopia. Visual acuity was better than 0.15 logMAR in both eyes in the control participants and the strabismus without amblyopia group. Amblyopia was defined as subnormal corrected distance visual acuity in the absence of any structural optic nerve, retinal, or visual pathway abnormalities. All subjects categorized within the amblyopic cohort exhibited at least a 2-line difference in visual acuity between the two eyes at the time of diagnosis. The inclusion criteria for amblyopic subjects were specified as the presence of amblyopia attributable to anisometropia or those with strabismus and mixed amblyopia defined per previous PEDIG studies [30]. The exclusion criteria included any coexisting ocular or systemic disease, congenital infections or malformations, developmental delay, or bilateral amblyopia defined as visual acuity in both eyes worse than 0.3 logMAR [31]. We also excluded subjects with Idiopathic Infantile Nystagmus, characterized by an increasing velocity slow-phase, or alternatively, pendular nystagmus, which can affect visual acuity [32].

### 2.2. Visual Acuity and Stereoacuity Measurements

Psykinematix v1.9.4 (KyberVision, Sendai, Japan) software was used to generate test stimuli on a 1280 × 800 resolution monitor at 60 Hz, with a white luminance of 111 cd/m^2^, viewed by subjects from 3.1 m in a dark room. Visual acuity was measured under binocular and monocular (right and left eye) viewing, with the non-viewing eye occluded. We conducted one trial for each of the three viewing conditions. Subjects viewed a randomly selected ETDRS optotype with crowding bars. This method adjusts the optotype size based on the subject’s responses using a 2-down-1-up staircase approach. The step size decreases by 50% before the first reversal and then changes by 25% increments and 12.5% decrements, for a total of six reversals, and calculations in arcmin are then converted to logMAR for analysis. The order of visual acuity trials under monocular and binocular viewing conditions was randomized.

For amblyopic subjects, the amblyopic eye was designated as the non-dominant eye, and the fellow eye was designated as the dominant eye. For the controls and strabismic subjects without amblyopia, the better monocular acuity eye was designated the dominant eye, and the worse eye as the non-dominant eye. Binocular performance of visual acuity was assessed by calculating the ratio of fellow/dominant eye acuity to binocular acuity, where a ratio of >1 indicates binocular summation and a ratio of <1 indicates binocular inhibition [32]. Stereoacuity was measured using the Titmus test in log arcsec at the time of recruitment of the study participants. Those with nil stereopsis were assigned a value of 3.85 log arcsec.

### 2.3. Fixational Eye Movements

A high-resolution eye tracker (EyeLink 1000, SR Research, Kanata, ON, Canada) was used to record horizontal and vertical eye positions of both eyes in binocular and monocular (right/left and fellow/amblyopic) eye viewing. If applicable, subjects wore their corrective lenses during eye movement recordings. The subject’s head was supported 84 cm away from the LCD screen on a chinrest. A 5-point constellation of targets was used to calibrate and validate each eye. The subjects fixated their gaze on a white circular target (0.5 degrees visual angle) projected against a black background on the LCD 30-inch monitor with a resolution of 2560 × 1600 at 60 Hz with brightness of 350 cd/m^2^. The recordings were obtained in a dark room under conditions of both eye viewing and monocular (fellow and amblyopic eye) viewing conditions. One trial was conducted for each of the three viewing conditions, and each trial lasted for 45 s. For monocular conditions, an infrared permissive filter was used to block visible light, enabling the recordings of eye movements of the covered (non-viewing) eye (open-loop testing). The order of the trials was randomized and the trials were conducted during the same visit as the psychophysical visual acuity measurements.

Using MatLab (MathWorks, Natick, MA, USA, https://www.mathworks.com), the subject’s eye positions were recorded and analyzed as a time series (position vs. time) after first identifying and removing the duration of the recordings that included blinks and partial blinks. Fusion Maldevelopment Nystagmus was diagnosed based on two specific criteria [30,31,32,33]: a slow-phase drift directed nasally relative to the fixating eye, and an immediate reversal of the fast and slow phases when the viewing eye was switched from one monocular viewing to another (dominant to non-dominant eye viewing, or vice versa). Nystagmus was identified in subjects who had repetitive sequences of a fast saccadic movement followed by a slower, linear, or decelerating drift during attempted fixation but did not fit the criteria for FMN.

We systematically evaluated fixation eye positions for all amblyopic/strabismic subjects and grouped them based on FEM waveforms into (a) controls, (b) those without nystagmus = 49 (anisometropic amblyopia = 22, strabismic amblyopia = 5, mixed amblyopia = 9, and strabismus without amblyopia = 13), (c) those with Fusion Maldevelopment Nystagmus (FMN) = 9 (anisometropic amblyopia = 0, strabismic amblyopia = 6, Mixed = 2, and strabismus without amblyopia = 1), and (d) those with other nystagmus (that did not meet the criteria of FMN or Idiopathic Infantile Nystagmus) = 27 (anisometropic amblyopia = 6, strabismic amblyopia = 9, mixed amblyopia = 9, and strabismus without amblyopia = 3). Due to the limited sample size, for the purpose of analysis, we combined subjects with Fusion Maldevelopment Nystagmus and other nystagmus together into the nystagmus group.

### 2.4. Statistical Analysis

Statistical analysis was conducted using SPSS (Version 25). Age and visual acuity differences between the control and amblyopic groups were compared using an unpaired *t*-test, while gender and race distributions were assessed with a chi-square test. Data normality was checked with the Kolmogorov–Smirnov test. Visual acuity per type and waveform was analyzed using two-way repeated measures ANOVA (one within viewing conditions and one using between-subject factors—clinical subgroups or FEM subgroups). For all the tests, Levene’s test of equality of error variances and Mauchly’s test of sphericity were conducted to test the assumption of homogeneity of variance. Mauchly’s test of sphericity indicated that the assumption of sphericity had been violated for the two-way interaction when comparing visual acuity per clinical group (χ^2^(2) = 121.7, *p* < 0.001) and per FEM group (χ^2^(2) = 138.18, *p* < 0.001), and Greenhouse–Geisser correction was also used. Binocular performance ratios were computed and compared across type and FEM waveform groups using one-way ANOVA.

Fixation instability was analyzed using three-way repeated measures ANOVA (two within-subject factors—(a) viewing conditions: binocular viewing, fellow/dominant eye viewing, and amblyopic/non-dominant eye viewing and (b) eye: fellow/dominant eye and amblyopic/non-dominant eye, and one between-subject factors—clinical subgroup). Mauchly’s test of sphericity indicated that the assumption of sphericity had been violated for the two-way interaction when comparing fixation instability per clinical group (χ^2^(2) = 6.82, *p* = 0.03), and Greenhouse–Geisser correction was used. Data are mean ± standard deviation unless otherwise stated.

The frequency of fast FEMs was analyzed using two-way repeated measures ANOVA (one within-subject factors—viewing conditions: binocular viewing, fellow/dominant eye viewing, and amblyopic/non-dominant eye viewing, and one between-subject factors—FEM subgroup). The velocity of slow FEMs was analyzed using three-way repeated measures ANOVA (two within-subject factors—(a) viewing conditions: binocular viewing, fellow/dominant eye viewing, and amblyopic/non-dominant eye viewing and (b) eye, and one between-subject factors—FEM subgroup). Mauchly’s test of sphericity indicated that the assumption of sphericity had been violated for the two-way interaction when comparing fixation instability per clinical group (χ^2^(2) = 121.85, *p* < 0.001), and Greenhouse–Geisser correction was used. Data are mean ± standard deviation unless otherwise stated.

Hierarchical multiple regression analyses were conducted to determine the factors influencing binocular and fellow eye visual acuity deficits. In the first model, we evaluated binocular visual acuity (dependent variable) as a function of continuous independent variables; visual acuity deficit of the amblyopic (AE) and fellow eye (FE) in log MAR, binocular performance ratio of visual acuity (ratio of FE visual acuity and binocular visual acuity), clinically measured strabismus angle in prism diopters (Δ), the extent of anisometropia (difference of spherical equivalent of refractive errors between the two eyes in diopter), age, stereoacuity deficits in log arc sec, fixation instability of AE and FE under binocular viewing in log BCEA, and multi-categorical waveform characteristics (whether subjects were with or without nystagmus, denoted by binary coding). In the second model, multi-categorical waveform variable information was removed.

For the hierarchical multiple regression, we reported the standardized coefficients, which provide the individual regression coefficients for each predictor variable that are standardized to allow for a comparison of the relative importance of each predictor. Larger standardized coefficients (β) in a regression model indicate a stronger and more important predictor variable. A negative standardized coefficient indicates a negative relationship between the predictor and the dependent variable. *R*^2^, or the coefficient of determination, indicates the proportion of variance in the dependent variable explained by the independent variable, with greater values representing a better fit of the model. A higher F value with concomitant *p* value < 0.05 indicates that the overall regression model is statistically significant and that the model is better with predictors. We determined the change in Model 1 and Model 2 performance by calculating changes in *R*^2^ (∆*R*^2^) and *F* (∆*F*). We repeated this regression model utilizing fellow eye visual acuity as the dependent variable.

To ensure model assumptions were met, several diagnostics were performed. The independence of residuals was validated by Durbin–Watson statistics, which remained below 2.4 across the models. No issues of multicollinearity were detected, with tolerance values exceeding 0.1 and variance inflation factors (VIFs) below 3. Additionally, no studentized deleted residuals exceeded ± 3 standard deviations. All statistically significant results had a critical alpha value of less than 0.05.

## 3. Results

Demographics (age, sex, and race) were collected via self-report for all study participants. The amblyopia/strabismus group had a mean age of 12.7 ± 11.3 years (55.3% female, 44.7% male), and the control group had a mean age of 12.1 ± 9.6 years (54.2% female, 45.8% male). There were no significant differences in age (*p* = 0.79) or gender (*p* = 0.92) between the two groups. There was no difference in the distribution of race between the controls (16 Caucasian [66.7%]; 3 African American [12.5%]; 1 Hispanic [4.2%]; 2 Asian [8.3%]; and 2 Multi-cultural [8.3%]) and amblyopia/strabismus subjects (58 Caucasian [68.2%]; 7 African American [8.3%]; 11 Hispanic [12.9%]; 3 Asian [3.5%]; 5 Multi-cultural [5.9%]; and 1 American Indian [1.2%]) (*p* = 0.88).

### 3.1. Binocular, Fellow/Dominant Eye, and Amblyopic/Non-Dominant Eye Visual Acuity per the Clinical Subtype

**Figure 1:**
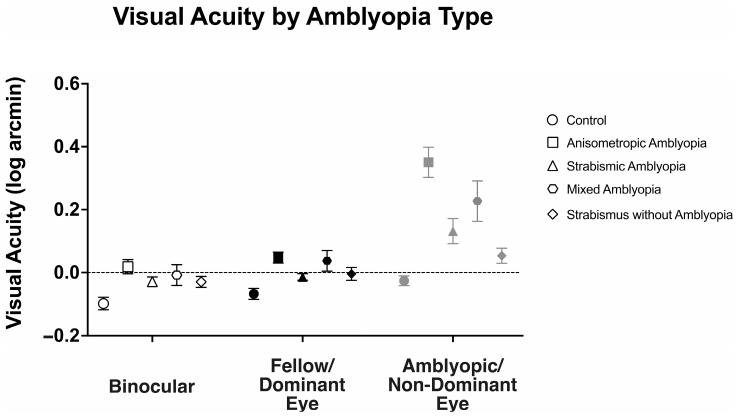
Mean (±standard error mean) binocular, fellow/dominant eye, and amblyopic/non-dominant eye visual acuity (log arcmin) per clinical subtype of amblyopia.

We examined the visual acuity of anisometropic, strabismic, and mixed amblyopia subjects, strabismus without amblyopia, and the control subjects (Figure 1). There was a statistically significant interaction between viewing conditions (binocular, fellow/dominant eye, and amblyopic/non-dominant eye viewing) across different groups, F(4.87, 126.73) = 15.49, *p* < 0.001, *η*^2^*_p_* = 0.37.

Binocular visual acuity (logMAR) was significantly affected by the presence of amblyopia (controls: −0.10 ± 0.10, anisometropic amblyopia: 0.02 ± 0.12, strabismic amblyopia: 0.04 ± 0.12, mixed amblyopia: 0.0 ± 0.08, and strabismus without amblyopia: −0.03 ± 0.08, F(4104) = 6.14, *p* < 0.001, *η*^2^*_p_* = 0.19). Anisometropic (*p* = 0.001), strabismic (*p* < 0.001), and mixed (*p* = 0.02) amblyopia subjects demonstrated worse binocular visual acuity than the controls, while strabismus without amblyopia subjects demonstrated similar binocular visual acuity compared to the controls (*p* = 0.46).

Similarly, fellow/dominant eye visual acuity was significantly affected by the presence of amblyopia (controls: −0.07 ± 0.08, anisometropic: 0.05 ± 0.09, strabismic: 0.07 ± 0.10, mixed: 0.03 ± 0.14, and strabismus without amblyopia: −0.01 ± 0.08, F(4104) = 6.83, *p* < 0.001, *η*^2^*_p_* = 0.21). Anisometropic (*p* = 0.001), strabismic (*p* < 0.001), and mixed (*p* = 0.02) amblyopia subjects demonstrated worse fellow eye visual acuity than the controls, while strabismus without amblyopia subjects demonstrated similar binocular visual acuity compared to the controls (*p* = 0.84).

As expected, amblyopic/non-dominant eye visual acuity deficits were significantly affected by presence of amblyopia (controls: −0.03 ± 0.08, anisometropic: 0.35 ± 0.25, strabismic: 0.25 ± 0.19, mixed: 0.50 ± 0.33, and strabismus without amblyopia: 0.04 ± 0.10, F(4104) = 21.89, *p* < 0.001, *η*^2^*_p_* = 0.46). Anisometropic (*p* < 0.001), strabismic (*p* < 0.001), and mixed (*p* < 0.001) amblyopia subjects demonstrated worse fellow eye visual acuity than the controls, while strabismus without amblyopia subjects demonstrated similar binocular visual acuity compared to the controls (*p* = 1.00).

**Figure 2:**
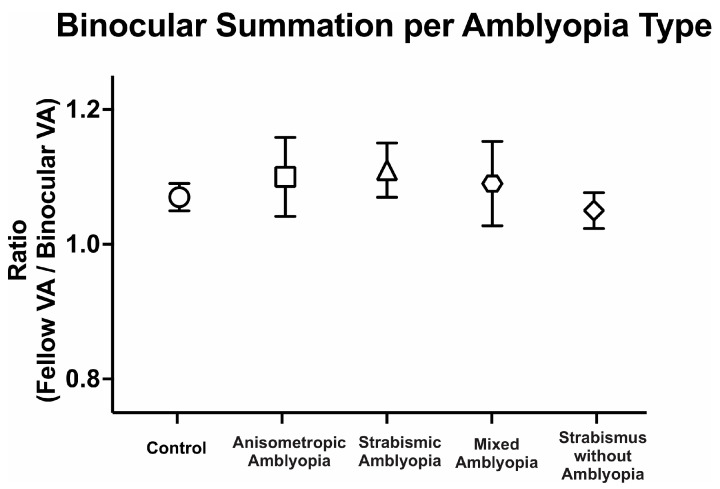
Mean (±standard error mean) binocular summation ratio [fellow/dominant eye visual acuity (VA; arcmin) divided by binocular VA] per clinical subtype of amblyopia.

We then compared binocular performance of visual acuity by calculating the ratio of fellow or dominant eye visual acuity in arcmin divided by binocular visual acuity arcmin. We evaluated the binocular performance ratios per the clinical type, with ratios greater than 1 signifying better binocular than fellow eye visual acuity, i.e., binocular summation, and ratios <1 signifying binocular inhibition. There was no difference in binocular performance ratios between the controls, amblyopic, and strabismus subjects (controls: 1.08 ± 0.10, anisometropic amblyopia: 1.10 ± 0.31, strabismic amblyopia: 1.11 ± 0.18, mixed amblyopia: 1.09 ± 0.28, and strabismus without amblyopia: 1.05 ± 0.11, F(4, 104) = 0.17, *p* = 0.95, *η*^2^*_p_* = 0.01). Despite the reduction in binocular acuity, amblyopic and strabismic subjects without amblyopia exhibited statistically comparable binocular performance ratios to the controls (Figure 2).

Thus, amblyopic subjects have worse visual acuity in the amblyopic eye as expected but also have worse binocular visual acuity and fellow eye visual acuity compared to the controls. On the other hand, strabismus without amblyopia subjects have slightly lower visual acuity in the binocular, dominant, and non-dominant eye compared to the controls but did not reach statistical significance. Additionally, amblyopia subjects, regardless of the presence of concurrent strabismus, and strabismic subjects demonstrate comparable binocular summation of visual acuity to the controls.

### 3.2. Fixation Instability Under Different Viewing Conditions per the Clinical Subtype

**Figure 3:**
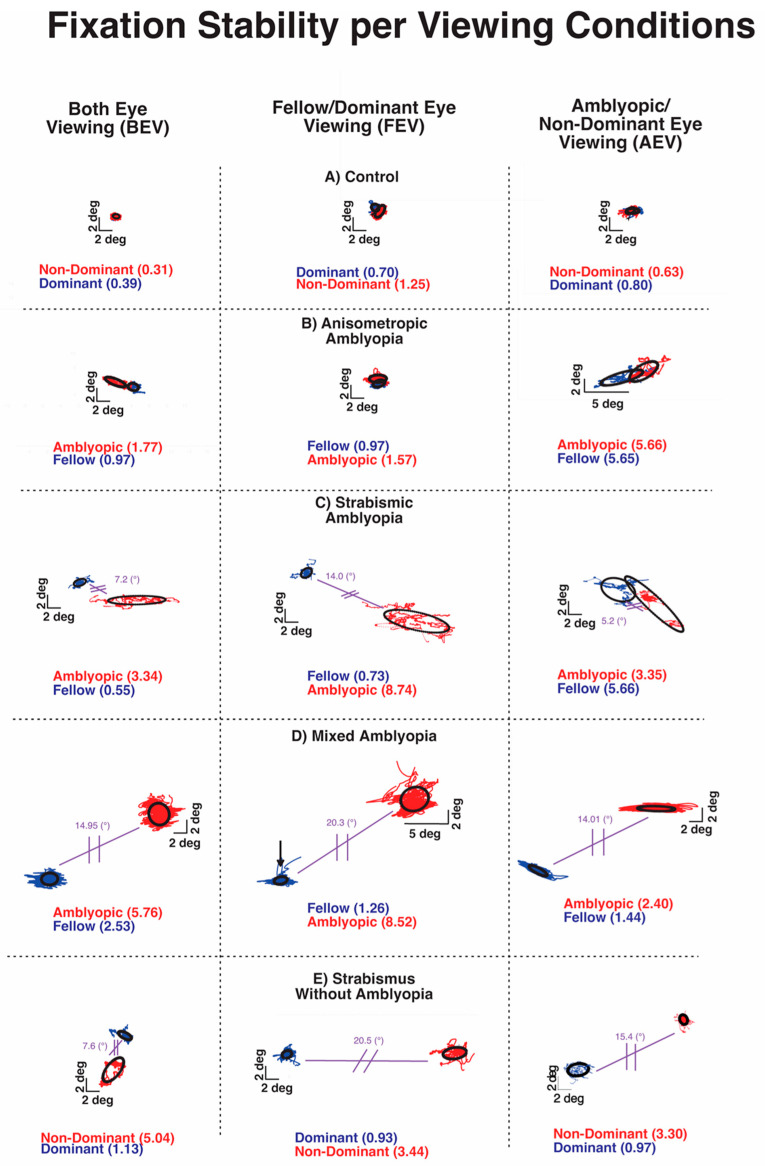
Examples of fixation instability under both eye viewing (BEV), fellow/dominant eye viewing (FEV), and amblyopic/non-dominant eye viewing (AEV) from a control subject (**A**), subject with anisometropic amblyopia (**B**), subject with strabismic amblyopia (**C**), subject with mixed amblyopia (**D**), and subject with strabismus without amblyopia (**E**) of the amblyopic/non-dominant eye (red) or fellow/dominant eye (blue). Black circles depict the logarithm of the bivariate contour ellipse area (logBCEA) that encompassed 68% of fixation points. Values depict the BCEA (deg^2^) of each eye in every viewing condition. The composite strabismic angle is depicted in purple.

Previous studies from our and other labs have shown increased fixation instability in amblyopia and strabismus without amblyopia [24,26,33]. Figure 3 plots the closed-loop (with visual feedback) and open-loop (without visual feedback) fixation instability (FI) in a control subject and subjects with anisometropic, strabismic and mixed amblyopia, and strabismus without amblyopia. The three boxes of the row depict BCEA plots and logBCEA values during binocular viewing, fellow eye/dominant eye viewing, and amblyopic eye/non-dominant eye viewing, respectively. The numbers within each box represent the calculated 68% value, which represents the area within which 68% of fixation points fall, as measured by the BCEA for each eye. Lower logBCEA numbers equate to more stability and a smaller area of the BCEA plot (negative values occur due to logarithmic transformation); higher numbers equate to more instability and a greater area of the plot.

All of the amblyopic subjects of Figure 3 had larger BCEA than the control subjects, indicating greater fixation instability. The increased FI was apparent in the fellow eye and amblyopic eye with greater instability in the amblyopic eye during BEV (left column) in each type of amblyopia. Fixation instability was also observed in strabismic subjects without amblyopia. Overall, fixation instability without visual feedback (open-loop condition) in the amblyopic/non-dominant eye was much greater in subjects with coexisting strabismus (Figure 3C–E).

The FI of the control subject did increase without visual feedback, attributable to minor instability caused by lack of binocular summation and elimination of binocular-disparity eye position feedback. All amblyopic subjects and strabismic amblyopia subjects also exhibited an increase in FI monocular viewing than binocular viewing. However, the characteristics of FI differ with greater instability observed in closed-loop FI of AE in AEV than in closed-loop FI of FE in FEV in all three types of amblyopia. On the other hand, in strabismic subjects without amblyopia, there was no such relative increase in closed-loop FI of the non-dominant eye in AEV than the dominant eye in the FEV condition. Also, the open-loop FI was markedly higher in amblyopic subjects with strabismus (Figure 3C,D) and strabismic subjects without amblyopia (Figure 3E).

**Figure 4:**
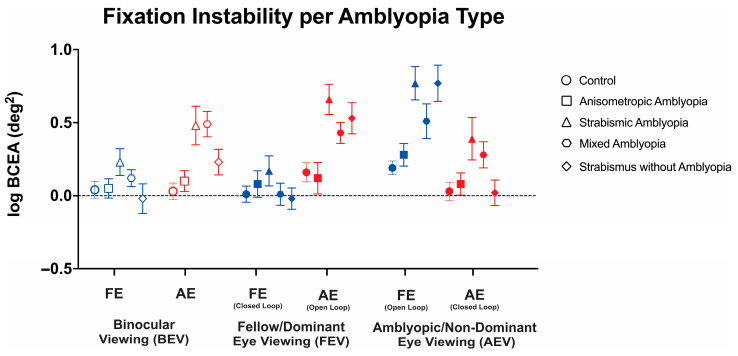
Mean (±standard error mean) logBCEA fixation instability (deg^2^) of the fellow/dominant eye (FE) and amblyopic/non-dominant eye (AE) per clinical subtype of amblyopia. Fixation instability (FI) is measured in both fellow eye (FE) and amblyopic eye (AE) in binocular viewing (BEV). In monocular viewing, closed-loop FI refers to the fixation instability of the viewing eye, while open-loop FI refers to the FI of the non-viewing (occluded) eye.

Thus, we conducted a three-way ANOVA to evaluate the changes in FI with or without visual feedback across these three viewing conditions in the controls, anisometropic amblyopia, strabismic amblyopia, mixed amblyopia, and strabismus without amblyopia (Figure 4). We found a statistically significant three-way interaction between clinical subtype, viewing condition, and eye (F(7.95, 206.62) = 4.95, *p* < 0.001, *η*^2^*_p_* = 0.1). Therefore, simple main effects and two-way interactions were run. The main effect of the viewing condition showed a statistically significant difference with the greatest instability observed in amblyopic/non-dominant eye viewing (F(2, 208) = 6.18, *p* = 0.002, *η*^2^*_p_* = 0.11). The main effect of the eye showed a statistically significant difference with greater FI in the amblyopic eye than fellow eye (F(1104) = 18.82, *p* < 0.001). There was a statistically significant two-way interaction between the clinical subtype and eye (F(4, 104) = 5.87, *p* < 0.001, *η*^2^*_p_* = 0.18). There was a statistically significant two-way interaction between viewing condition and eye (F(1.99, 206.62) = 83.84, *p* < 0.001, *η*^2^*_p_* = 0.45). Strabismic and mixed amblyopia subjects demonstrated worse fixation stability of the amblyopic/non-dominant eye in binocular viewing than the controls and anisometropic amblyopia subjects (control: logBCEA 0.03 ± 0.28, anisometropic: 0.05 ± 0.35, strabismic: 0.48 ± 0.59, mixed: 0.49 ± 0.39, and strabismus without amblyopia: 0.48 ± 0.59, F(4, 104) = 6.11, *p* < 0.001, *η*^2^*_p_* = 0.19). No significant differences were observed in fellow eyes in binocular viewing (F(4, 104) = 1.54, *p* = 0.20, *η*^2^*_p_* = 0.06).

Strabismic amblyopia subjects demonstrated worse open-loop fixation stability in fellow/dominant eye viewing than the controls and anisometropic amblyopia subjects (F(4, 104) = 6.32, *p* < 0.001, *η*^2^*_p_* = 0.20). No significant differences were observed in closed-loop FI in fellow eye viewing (F(4, 104) = 0.79, *p* = 0.54, *η*^2^*_p_* = 0.03).

In amblyopic/non-dominant eye viewing, strabismic amblyopia subjects demonstrated worse closed-loop (F(4, 104) = 6.19, *p* < 0.001, *η*^2^*_p_* = 0.19) and open-loop (F(4, 104) = 3.03, *p* = 0.02, *η*^2^*_p_* = 0.10) FI compared to the controls and anisometropic amblyopia patients.

Thus, amblyopic subjects with strabismus (strabismic/mixed amblyopia) demonstrate worse fixation instability with (closed-loop) or without (open-loop) visual feedback with greater abnormalities observed in the amblyopic eye, whereas greater instability was observed in the non-viewing eye (open-loop) in strabismic without amblyopia subjects under monocular viewing.

### 3.3. Nystagmus as an Element of Fixation Instability Observed in Amblyopia and Strabismus Without Amblyopia

**Figure 5:**
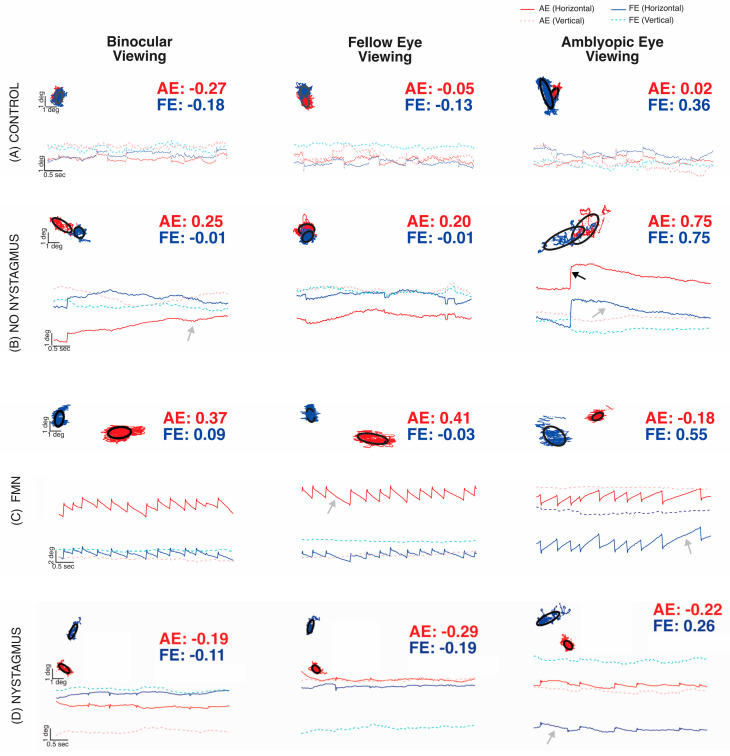
Examples of fixation eye movements under both eye viewing (BEV), fellow eye viewing (FEV), and amblyopic eye viewing (AEV) conditions from a control subject (**A**), subject without nystagmus (**B**), subject with Fusion Maldevelopment Nystagmus (**C**), and subject with other nystagmus (**D**). The *x*-axis represents time, and the *y*-axis represents horizontal (solid line, dark blue: fellow/dominant eye, red: amblyopic/non-dominant eye) and vertical (dotted line, pink: fellow eye, light blue: amblyopic eye) positions. Rightward and upward movements correspond to the positive vertical axis. Each plot contains the BCEA scatter plot with the black circles depicting the logarithm of the bivariate contour ellipse area (logBCEA) that encompassed 68% of fixation points with the corresponding logBCEA values of the amblyopic/non-dominant eye (red) or fellow/dominant eye (blue).

In this study, fixation instability (FI) as measured by the bivariate contour ellipse area (BCEA) was higher in all amblyopic subjects and strabismic subjects compared to the control values. These instabilities worsened when subjects were deprived of binocular vision cues, indicating that FI was exacerbated during monocular viewing. Despite its usefulness, BCEA does not capture the dynamic characteristics of fixation movements (FEMs) in some amblyopes, such as fixational nystagmus. Additionally, clinical examinations may not always accurately detect nystagmus. Accurate detection of nystagmus requires careful analysis of eye movement recordings, focusing on fast and slow-phase oscillations. To identify nystagmus, eye position traces were analyzed during fixation with or without visual feedback (Figure 5). Subjects were then categorized based on the presence or absence of nystagmus and its type: no nystagmus, other nystagmus, and Fusion Maldevelopment Nystagmus.

Figure 5A depicts epochs of fast and slow components of FEMs acquired from a control subject, whereas Figure 5B depicts an example of the amblyopic subject without nystagmus obtained during binocular viewing, FEV, or AEV conditions. The BCEA plots superimpose the horizontal (*x*-axis) and vertical (*y*-axis) eye positions of the right or amblyopic (red) vs. left or fellow (blue) eye. The control (Figure 5A) and amblyopia subject without nystagmus (Figure 5B) tracings show some fixational saccades interspersed with inter-saccadic drifts but lack repetitive oscillations. They were categorized as no nystagmus. Compared to the control subject, the amblyopic subject without nystagmus had an increased fixation instability with or without visual feedback, as well as an increase in the drift of the amblyopic eye. Figure 5C demonstrates an example of a subject with a conjugate horizontal jerk nystagmus in binocular viewing. Fellow eye viewing (right eye) showed equivalent, leftward slow phase nystagmus, whereas amblyopic eye viewing (left eye) produced an inversion of the nystagmus; slow phases were now directed rightward in both eyes at a higher velocity. The nystagmus was categorized as Fusion Maldevelopment Nystagmus, typified by a conjugate nasalward slow phase with respect to the viewing eye; rightward when viewing with the left AE, and leftward when viewing with the right FE. Figure 5D demonstrates an example of an amblyopic subject who had nystagmus, which is most evident in the amblyopic eye viewing condition. When viewing changed to FEV, the repetitive nystagmus cycles disappeared. We characterized this subject who did not meet the criteria of FMN or congenital motor nystagmus as a subject with other nystagmus. It is important to note that, in the two examples of subjects with nystagmus, BCEA values alone are unable to differentiate between amblyopic subjects with and without nystagmus [34].

### 3.4. Frequency of Fast FEMs and Velocity of Slow FEMs Under Binocular and Monocular Viewing per the Presence of Nystagmus

**Figure 6:**
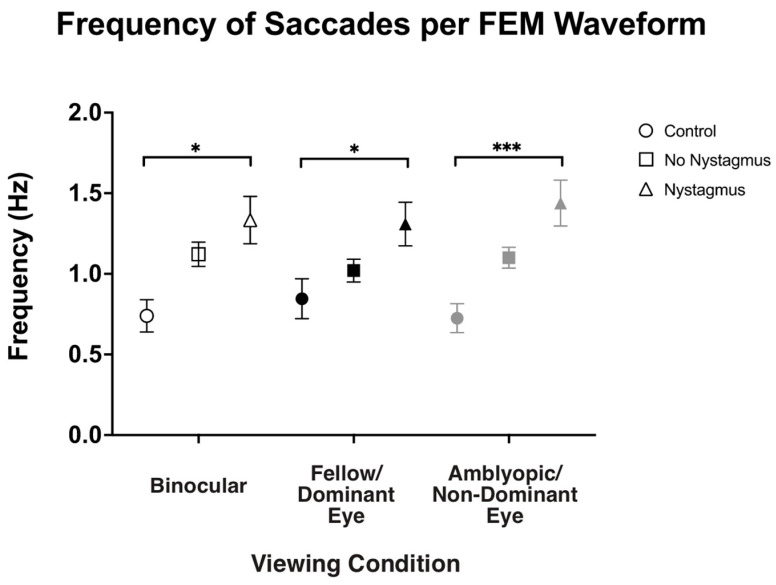
Median and standard error mean of the frequency of saccades (Hz) in binocular, fellow/dominant eye, and amblyopic/non-dominant eye visual acuity per Fixational Eye Movement (FEM) waveform subgroups. A single asterisk (*) denotes a *p*-value < 0.05 and triple asterisks (***) denote a *p*-value ≤ 0.001. The fellow/dominant eye is depicted in blue and the amblyopic/non-dominant eye is depicted in red.

Besides visual inspection, we also computed the frequency of fast FEMs (i.e., fixational saccades in the controls and subjects without nystagmus and quick phases in subjects with nystagmus) under different viewing conditions (Figure 6). As expected, the frequency of fast FEMs (Hz) in binocular viewing was significantly increased in amblyopic and strabismic subjects with nystagmus compared to the controls (controls: 0.71 ± 0.43, no nystagmus: 1.12 ± 0.51, and nystagmus: 1.32 ± 0.84, F(2,93) = 4.77, *p* = 0.01, *η*^2^*_p_* = 0.09), with post hoc pairwise comparisons demonstrating a *p*-value of 0.008. Similarly, in fellow/dominant eye viewing, the frequency of fast FEMs was increased in subjects with nystagmus (controls: 0.80 ± 0.47, no nystagmus: 1.03 ± 0.48, and nystagmus: 1.28 ± 0.78, F(2,93) = 3.70, *p* = 0.03, *η*^2^*_p_* = 0.07), with post hoc pairwise comparisons demonstrating a *p*-value of 0.034. Also, the fast FEM frequency under amblyopic/non-dominant eye viewing was also increased in subjects with nystagmus compared to the controls (controls: 0.70 ± 0.36, no nystagmus: 1.10 ± 0.43, and nystagmus: 1.43 ± 0.81, F(2106) = 16.25, *p* < 0.001, *η*^2^*_p_* = 0.24), with post hoc pairwise comparisons demonstrating a *p*-value < 0.001.

**Figure 7:**
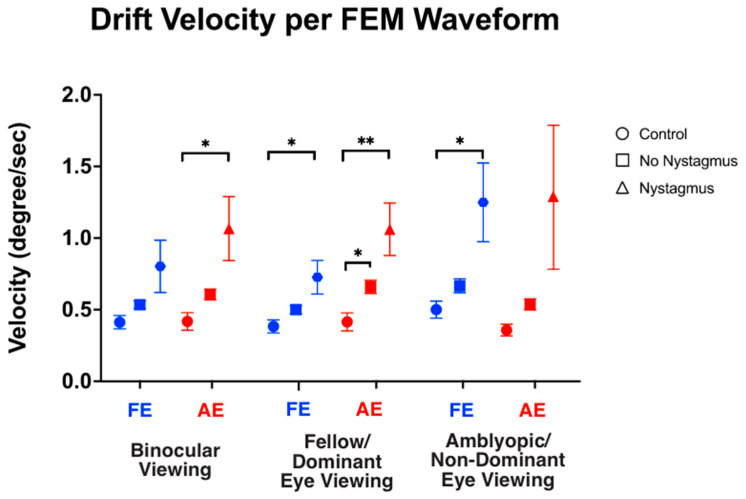
Median and standard error mean of the drift velocity (deg/sec) in binocular, fellow/dominant eye, and amblyopic/non-dominant eye visual acuity per Fixational Eye Movement (FEM) waveform subgroups. A single asterisk (*) denotes a *p*-value < 0.05 and double asterisks (**) denote a *p*-value ≤ 0.01. The fellow/dominant eye is depicted in blue and the amblyopic/non-dominant eye is depicted in red.

We also computed the velocity of slow FEMs (i.e., inter-saccadic drifts in the controls and subjects without nystagmus, and slow phase FEMs in subjects with nystagmus) under different viewing conditions (Figure 7). As expected, under binocular viewing, the slow FEM velocity was significantly increased in subjects with nystagmus compared to the controls in the amblyopic/non-dominant eye (controls: 0.43 ± 0.26, no nystagmus: 0.60 ± 0.25, and nystagmus: 1.06 ± 1.26, F(2,93) = 4.73, *p* = 0.01, *η*^2^*_p_* = 0.09), with post hoc pairwise comparisons demonstrating a *p*-value = 0.03. Although increased velocity was seen in the fellow/dominant eye of subjects with nystagmus, the values did not reach statistical significance (controls: 0.39 ± 0.19, no nystagmus: 0.54 ± 0.18, and nystagmus: 0.79 ± 1.04, F(2,93) = 2.50, *p* = 0.08, *η*^2^*_p_* = 0.05), with post hoc comparisons demonstrating a *p*-value = 0.13.

Under fellow/dominant eye viewing, the velocity was significantly increased in subjects with nystagmus compared to the controls in the viewing fellow eye (controls: 0.38 ± 0.19, no nystagmus: 0.50 ± 0.20, and nystagmus: 0.71 ± 0.67, F(2,93) = 2.60, *p* = 0.023, *η*^2^*_p_* = 0.08) with post hoc comparisons demonstrating *p* = 0.007. Velocity was also significantly increased in the non-viewing eye (controls: 0.40 ± 0.25, no nystagmus: 0.66 ± 0.31, and nystagmus: 1.05 ± 1.04, F(2,93) = 5.94, *p* = 0.004, *η*^2^*_p_* = 0.09), with post hoc comparisons demonstrating *p* = 0.04.

Under amblyopic/non-dominant eye viewing, the velocity was significantly increased in subjects with nystagmus compared to the controls in the non-viewing eye (controls: 0.46 ± 0.23, no nystagmus: 0.67 ± 0.32, and nystagmus: 1.23 ± 1.56, F(2,93) = 4.78, *p* = 0.01, *η*^2^*_p_* = 0.09) (post hoc *p* = 0.03). The velocity did not reach significance in the viewing eye (controls: 0.35 ± 0.15, no nystagmus: 0.54 ± 0.26, and nystagmus: 1.26 ± 2.84, F(2,93) = 2.313, *p* = 0.10, *η*^2^*_p_* = 0.05) (post hoc *p* = 0.26).

These findings suggest that subjects with nystagmus had an increased frequency of fast FEMs across all viewing conditions compared to the controls. We also find that the slow FEM velocities are greater in the fellow eye of subjects with nystagmus under fellow eye viewing with no difference seen in the velocity of slow FMs in the fellow eye in binocular viewing.

### 3.5. Visual Acuity Under Binocular and Monocular Viewing per the Presence of Nystagmus

**Figure 8:**
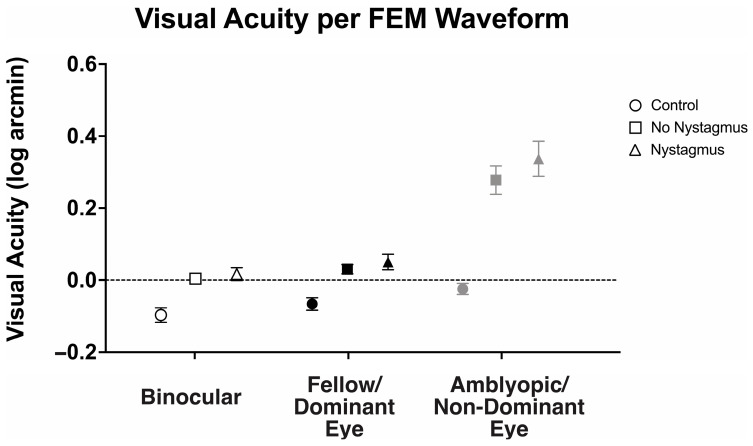
Mean and standard error mean of the visual acuity (logMAR) in binocular, fellow/dominant eye, and amblyopic/non-dominant eye visual acuity per Fixational Eye Movement (FEM) waveform subgroups.

With an understanding that visual motor deficits such as fixation instability can affect visual acuity, we then evaluated how the presence of nystagmus (FEM subgroup) affected the observed binocular and fellow eye visual acuity deficit (Figure 8). There was a statistically significant interaction between the viewing condition and FEM waveform groups, F(2.31, 122.42) = 7.98, *p* < 0.001, *η*^2^*_p_* = 0.13.

Binocular visual acuity (logMAR) was significantly worse in amblyopic and strabismic subjects with or without nystagmus, with the greatest abnormalities seen in those with nystagmus (controls: −0.10 ± 0.10, no nystagmus: 0.00 ± 0.10, and nystagmus: 0.02 ± 0.11, F(2106) = 7.80, *p* < 0.001, *η*^2^*_p_* = 0.16). Similarly, fellow eye visual acuity was worse in amblyopic and strabismic subjects with nystagmus and without nystagmus, with the greatest abnormalities seen in those with nystagmus (controls: −0.07 ± 0.08, no nystagmus: 0.03 ± 0.09, and nystagmus: 0.05 ± 0.13, F(2106) = 10.09, *p* < 0.001, *η*^2^*_p_* = 0.16). As expected, amblyopic/non-dominant eye visual acuity was also worse in amblyopic and strabismic subjects with or without nystagmus than the controls (controls: −0.03 ± 0.08, no nystagmus: 0.28 ± 0.28, and nystagmus: 0.34 ± 0.29, F(2106) = 16.25, *p* < 0.001, *η*^2^*_p_* = 0.24), but it was most pronounced in nystagmus.

Amblyopic subjects, both with or without nystagmus, demonstrated significant binocular summation. Binocular visual acuity (0.02 ± 0.11 logMAR) of subjects with nystagmus was significantly improved compared to monocular fellow eye acuity (0.05 ± 0.13) and amblyopic eye acuity (0.34 ± 0.29), F(2, 105) = 31.61, *p* < 0.001, *η*^2^*_p_* = 0.38, with mean differences of −0.03 (95% CI, −0.07 to −0.01) logMAR and −0.32 (95% CI, −0.42 to −0.22), respectively. Amblyopia subjects without nystagmus demonstrated significantly improved binocular visual acuity (0.00 ± 0.10 logMAR) compared to amblyopic eye acuity (0.28 ± 0.28), but the improvement did not reach statistical significance in fellow eye acuity (0.05 ± 0.13), F(2, 105) = 31.23, *p* < 0.001, *η*^2^*_p_* = 0.37, with mean differences of −0.27 (95% CI, −0.19 to −0.05) logMAR and −0.03 (95% CI, −0.05 to 0.00), respectively.

**Figure 9:**
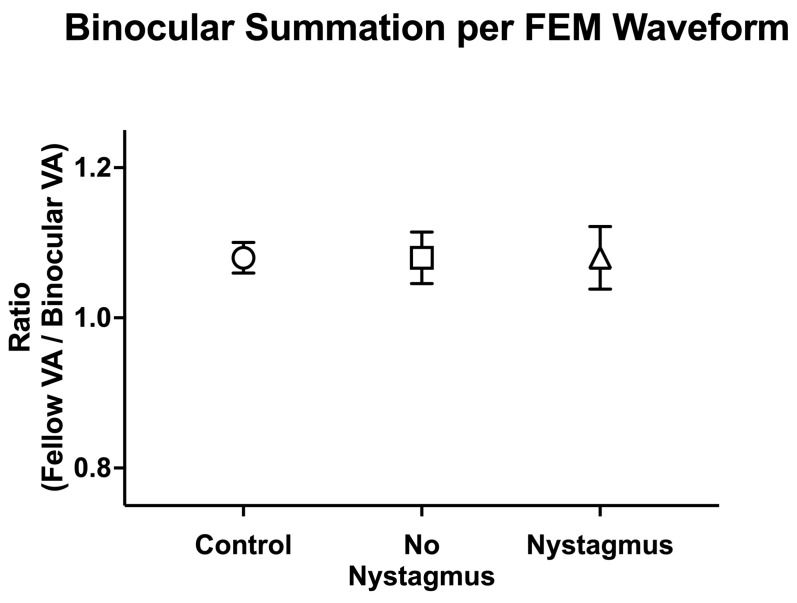
Mean (±standard error mean) binocular summation ratio [fellow/dominant eye visual acuity (VA; arcmin) divided by binocular VA] per Fixational Eye Movement (FEM) waveform subgroups.

We then compared binocular performance ratios (fellow/dominant eye logMAR VA divided by binocular logMAR VA) per the FEM waveform, with ratios greater than 1 signifying better binocular than fellow eye VA (Figure 9). There was no difference in binocular performance ratios between the controls, amblyopic, and strabismus subjects (controls: 1.08 ± 0.10, no nystagmus: 1.08 ± 0.24, and nystagmus: 1.10 ± 0.25, F(2106) = 0.12, *p* = 0.89, *η*^2^*_p_* = 0.002).

Thus, amblyopic and strabismus subjects with or without nystagmus have worse binocular and fellow eye visual acuity compared to the controls. Additionally, amblyopia subjects with concurrent strabismus with or without nystagmus demonstrate binocular summation, with improvements in visual acuity with binocular viewing compared to fellow eye/non-dominant eye viewing.

### 3.6. Impact of Fixation Instability, Nystagmus, and Visual Sensory Factors on Visual Acuity

We ran a hierarchical multiple regression analysis to evaluate how age, visual sensory (visual acuity and stereoacuity) and motor function abnormalities (fixation instability, nystagmus, and strabismus), and extent of anisometropia affect binocular visual acuity. In the first model, visual acuity of AE and FE, binocular performance ratio (ratio of FE and binocular visual acuity), clinically measured strabismus angle, the extent of anisometropia (refractive error difference), age, stereoacuity deficits, fixation instability, and multi-categorical waveform characteristics (with and without nystagmus) were included. This model statistically predicted binocular visual acuity (*R*^2^ = 0.99, Adj. *R*^2^ = 0.98, F(11,94) = 412.26, *p* < 0.001). In this model, greater fellow eye visual acuity deficit (β = 1.071, *p* < 0.001), reduced binocular summation (β = −0.681, *p* < 0.001), and increased FI of the amblyopic eye (β = 0.042, *p* = 0.046) were statistically significant factors that contributed to the prediction of binocular visual acuity deficits (Table 1). Larger standardized coefficients (β) in a regression model indicate a stronger and more important predictor variable. Interestingly, amblyopic eye visual acuity, stereoacuity deficit, strabismus angle, refractive error difference, and the presence of nystagmus did not significantly contribute to the prediction of binocular visual acuity deficit.

In the second model, multi-categorical waveform variable information was removed. This model also statistically predicted binocular visual acuity (*R*^2^ = 0.99, Adj. *R*^2^ = 0.98, F(2,94) = 514.14, *p* < 0.001). Similar to the first model, increased visual acuity deficit of the FE (β = 1.073, *p* < 0.001), reduced binocular summation (β = −0.682, *p* < 0.001), and increased FI of the amblyopic eye (β = 0.043, *p* = 0.039) were statistically significant factors that contributed to the prediction of binocular visual acuity deficits (Table 2). However, there was no statistically significant *R*^2^ and F change (Δ*R*^2^ = 0.00, ΔF = 0.41) after the removal of the waveform information in the second model (Table 1). In other words, the presence of nystagmus in the first model did not improve the ability of the model to predict the extent of binocular visual acuity deficit.

We then ran a hierarchical multiple regression analysis to evaluate how the presence of nystagmus and other clinical parameters affect fellow eye visual acuity (Table 2). In the first model, visual acuity of AE, clinically measured strabismus angle, the extent of anisometropia (refractive error difference), age, stereoacuity deficits, fixation instability of the fellow eye and fixation instability of the covered amblyopic eye (open-loop condition) without visual feedback, and multi-categorical waveform characteristics (in which subjects were categorized as “no nystagmus” or “nystagmus”) were included. This model statistically predicted fellow eye visual acuity (*R*^2^ = 0.40, Adj. *R*^2^ = 0.35, F(9,96) = 7.213, *p* < 0.001). In this model, greater amblyopic eye visual acuity deficit (β = 0.556, *p* < 0.001), greater refractive error difference (β = −0.377, *p* < 0.001), increased FI of the fellow eye (closed-loop condition) (β = 0.265, *p* = 0.014), waveform information, i.e., those without nystagmus (β = 0.293, *p* = 0.018), and those with nystagmus (β = 0.378, *p* = 0.009) were statistically significant factors that contributed to the prediction of fellow eye visual acuity deficits (Table 2).

In the second model, waveform variable information was removed. This model also statistically predicted binocular visual acuity (*R*^2^ = 0.36, Adj. *R*^2^ = 0.31, F(2,96) = 7.73, *p* < 0.001). In this model, greater amblyopic eye visual acuity deficit (β = 0.619, *p* < 0.001), refractive error difference (β = −0.311, *p* = 0.005), and increased FI of the fellow eye (β = 0.235, *p* = 0.030) were statistically significant factors that contributed to the prediction of fellow eye visual acuity deficits (Table 2). There was a statistically significant *R*^2^ and F change (Δ*R*^2^ = −0.05, ΔF = 3.83, *p* = 0.025) after the removal of the waveform information in the second model. In other words, the inclusion of the waveform variable in the first model did improve the ability of the model to predict the extent of fellow eye visual acuity deficit.

## 4. Discussion

This study is the first to examine binocular and fellow eye visual acuity and binocular summation across different clinical types of amblyopia and strabismus using psychophysical tasks. We observed significant visual acuity deficits of the fellow eye in amblyopic subjects, irrespective of the type of amblyopia. This finding is consistent with previous studies that have demonstrated fellow eye dysfunction in amblyopia [3]. In contrast, strabismic subjects without amblyopia showed smaller, non-significant reductions compared to the controls, suggesting that amblyopia—not strabismus alone—is primarily responsible for visual acuity deficits. We have previously shown that fixation instability of the fellow eye and amblyopic eye, measured with the bivariate contour ellipse area, varies across viewing conditions and is elevated during both fellow/dominant eye viewing and binocular viewing compared to the controls [27,35]. Hierarchical regression models revealed greater fellow eye acuity deficits in subjects with greater anisometropia, greater amblyopic eye visual acuity deficit, and greater fixation instability in the FE during fellow eye viewing (closed-loop or with visual feedback condition).

We also found reduced binocular visual acuity regardless of whether the subject had anisometropic, strabismic, or mixed mechanism clinical subtypes of amblyopia. Our hierarchical regression models showed that binocular visual acuity deficits also correlated with fellow/dominant eye acuity deficits and fixation instability of the amblyopic/non-dominant eye obtained during binocular viewing. Thus, our findings extend previous work linking fixation instability with visual acuity deficits in the amblyopic eye [24]. Our data supports a strong correlation between fixation instability and fellow eye and binocular visual acuity deficits in amblyopia, highlighting the bidirectional relationship between eye movement and visual acuity and acknowledging the complexity of determining causality. Interestingly, despite reduced fellow eye acuity, the binocular-to-fellow eye acuity ratio remained close to 1 across groups (with binocular visual acuity exceeding fellow/dominant eye visual acuity), indicating preserved binocular summation in amblyopia and strabismus. However, the hierarchical models showed that subjects with greater binocular acuity deficits had less binocular summation, adding to studies attributing reduced binocular contrast sensitivity in amblyopia to impaired summation [32,36].

Previous studies have shown that amblyopic eye visual acuity deficits are correlated with stereoacuity deficits. Further, even when the visual acuity deficit in the amblyopic eye resolves, amblyopic subjects often continue to experience persistent stereoacuity deficits [37]. This motivated us to investigate whether stereoacuity deficits also impact fellow and binocular visual acuity deficits. Interestingly, in our study, stereoacuity did not correlate with measured binocular and fellow eye visual acuity. However, stereopsis was assessed using the Titmus Fly Test, which, while commonly used, may yield false results due to the influence of monocular and non-stereoscopic binocular cues [38]. Thus, future studies that examine stereopsis using a rigorous psychophysical test are warranted.

Although BCEA quantifies fixation instability by quantifying eye position dispersion, it does not reliably detect nystagmus [34]. Determining the presence of nystagmus is important as previous studies have shown that nystagmus is influenced by viewing conditions, which impacts visual acuity. For instance, in Fusion Maldevelopment Nystagmus, occluding one eye worsens the nystagmus and reduces visual acuity in the non-occluded eye, while fogging the eye enhances acuity and improves nystagmus [29]. Therefore, we analyzed eye position traces to characterize nystagmus and compute frequencies of fast FEMs and velocities of slow FEMs in subjects with and without nystagmus. Velocities of slow FEMs of the fellow eye were significantly greater during fellow eye viewing in subjects with nystagmus compared to the controls. Hierarchical regression showed that adding nystagmus waveform data improved the model’s prediction of fellow eye acuity deficits, which is likely due to greater FEM abnormalities seen in subjects with nystagmus. This aligns with prior findings that nystagmus worsens contrast sensitivity at both low and high spatial frequencies in the fellow eye [35,39].

On the other hand, adding nystagmus waveform data did not significantly enhance the model’s prediction of binocular acuity deficits, suggesting that these deficits are present regardless of nystagmus. This could be attributed to more variability in the slow phase velocities in subjects with nystagmus during binocular viewing compared to fellow eye viewing, due to the dampening of nystagmus in binocular viewing. Additionally, amblyopic subjects without nystagmus often have alterations in physiologic FEMs in both eyes compared to the controls—explaining the association between reduced binocular acuity and increased fixation instability of the amblyopic eye in binocular viewing [24,25,33,34]. Interestingly, we found that binocular summation was similar in subjects with and without nystagmus—likely due to a dampening effect of binocular viewing on nystagmus.

In summary, this study is among the first to assess fixation eye movement (FEM) abnormalities and binocular and fellow eye acuity deficits in amblyopia with and without strabismus. We found that amblyopic subjects, regardless of strabismus or nystagmus, had lower binocular and fellow eye acuity than the controls, with the most severe deficits in those with nystagmus. Overall, these results highlight the complex interplay between amblyopia, strabismus, visual acuity deficits, fixation instability, and nystagmus. Thus, our findings emphasize the importance of eye movement evaluation to understand visual deficits in amblyopia and strabismus. Furthermore, our data underscore how viewing conditions affect both sensory and motor deficits in amblyopia and strabismus, emphasizing their close relationship. Limitations include a small sample size and lack of deprivation amblyopia cases, which restricted analysis of FEM waveforms across subtypes. Future studies with larger cohorts are needed to assess how FEM abnormalities affect visual acuity outcomes and to evaluate both traditional and novel amblyopia treatments.

## Figures and Tables

**Table 1:** Evaluation of factors impacting binocular visual acuity deficit using a hierarchical regression model.

	Model 1 with Waveform	Model 2 without Waveform
Constant	0.332	0.333
FE visual acuity	1.071 ***	1.073 ***
AE visual acuity	−0.014	−0.013
Binocular summation ratio	−0.681 ***	−0.682 ***
Strabismus angle	−0.01	−0.008
Refractive error difference	0.03	0.029
Age	0.00	−0.001
Stereopsis	−0.015	−0.014
FI of FE in binocular viewing	−0.025	−0.025
FI of AE in binocular viewing	0.042 *	0.043 *
No nystagmus	0.007	NA
Nystagmus	0.007	NA
Adj. *R*^2^, F	0.98, 412.26 ***	0.98, 514.14 *** ∆*R*^2^, ∆F = 0, 0.41

Note: Standard coefficients (β) of predictors (continuous independent variables) utilized to predict binocular visual acuity (dependent variable) in a hierarchical multiple regression model. Predictors included fixation instability (FI) of the fellow eye (FE) and amblyopic eye (AE) with visual feedback (BCEA, deg^2^), visual acuity (VA, logMAR), binocular summation (logMAR fellow eye to binocular VA ratio, with >1 indicating binocular summation), strabismus angle (Δ), refractive error difference (diopter), stereopsis (log arcsec), and presence of nystagmus (via binary coding). The greater the magnitude of the coefficient, the greater the importance of the predictor; negative coefficients denote a negative relationship. A single asterisk (*) denotes a *p*-value < 0.05, and triple asterisks (***) denote a *p*-value ≤ 0.001. NA = not applicable due to predictors being removed in Model 2.

**Table 2:** Evaluation of factors impacting fellow/dominant eye visual acuity deficit using a hierarchical regression model.

	Model 1 with Waveform	Model 2 without Waveform
Constant	−0.044	−0.034
AE visual acuity	0.556 ***	0.619 ***
Strabismus angle	−0.032	0.051
Refractive error difference	−0.377 ***	−0.311 **
Age	−0.096	−0.091
Stereopsis	0.002	0.086
Closed-loop FI of FE in FEV	0.265 *	0.235 *
Open-loop FI of AE in FEV	−0.085	−0.035
No nystagmus	0.293 *	NA
Nystagmus	0.378 **	NA
Adj *R* ^2^, F	0.35, 7.213 ***	0.31, 7.734 *** ∆*R*^2^, ∆F = −0.048, 3.827 ***

Note: Standard coefficients (β) of predictors (continuous independent variables) utilized to predict fellow eye visual acuity (dependent variable) in a hierarchical multiple regression model. Predictors included fixation instability (FI) of the fellow eye (FE) and amblyopic eye (AE) with visual feedback (BCEA, deg^2^), visual acuity (VA, logMAR), binocular summation (logMAR fellow eye to binocular VA ratio, with >1 indicating binocular summation), strabismus angle (Δ), refractive error difference (diopter), stereopsis (log arcsec), and presence of nystagmus (via binary coding). The greater the magnitude of the coefficient, the greater the importance of the predictor; negative coefficients denote a negative relationship. A single asterisk (*) denotes a *p*-value < 0.05, double asterisks (**) denote a *p*-value ≤ 0.01, and triple asterisks (***) denote a *p*-value ≤ 0.001. NA = not applicable due to predictors being removed in Model 2.

## Data Availability

The raw data supporting the conclusions of this article will be made available by the authors upon request.

## Abbreviations

The following abbreviations are used in this manuscript:

AE	Amblyopic eye
AEV	Amblyopic eye viewing
BEV	Binocular viewing
FE	Fellow eye
FEV	Fellow eye viewing
FEM	Fixation eye movement
FI	Fixation instability
MV	Monocular viewing
VA	Visual acuity
